# What Are the Precipitating Factors of Suicide in South Korea? A Systematic Review

**DOI:** 10.1192/bjo.2024.146

**Published:** 2024-08-01

**Authors:** Fatima Gasimzade, Sian Marie Edney, George Kirov

**Affiliations:** Cardiff University, Cardiff, United Kingdom

## Abstract

**Aims:**

Today the problem of suicide remains one of the topical issues among Asian countries like South Korea. The primary objective of this Systematic Review (SR) was the identification of the risk factors in suicide attempts and completions in South Korea. South Korea has one of the highest reported suicide rates in the world, and there was no prior systematic review on this topic. The main intention was to provide evidence-based results for future research studies and to inform suicide prevention policies.

**Methods:**

PubMed, Science Direct, and Medline databases were searched from 1990 to October 2022. Studies focused on the problem of suicide in South Korea were selected with an emphasis on risk factors. Since the rate of suicide rises with age, studies examining 18-year-olds and above were included. Studies examining people of different sociodemographic backgrounds and people diagnosed with psychiatric/psychological disorders and those who attempted suicide, as well as those without a psychiatric/psychological problem, were also included. To assess the overall quality of the included studies, the Critical Appraisal Skills Programme (CASP) checklist was applied for the evaluation of case-control and cohort studies. The National Institutes of Health (NIH) Quality Assessment Tool was implemented for the evaluation of cross-sectional studies. The Jadad Scale was used to assess the risk of bias. PRISMA reporting guidelines were followed.

**Results:**

Ten studies met the eligibility criteria to be included in SR: Four cross-sectional, two cross-cultural, and four cohort studies. Risk of bias assessment demonstrated low quality and moderate risk of bias. The quality assessment showed an acceptable level of relevance and quality. Findings suggested factors leading to suicide in South Korea were mental health conditions, financial status, such as unemployment and low income, education level less than high school, and households with poor living conditions. The identified risk factors significantly increased the likelihood of suicide ideation, suicide attempt and suicide completion among people in South Korea. Stigma was identified as a barrier to those with mental health conditions seeking professional help.

**Conclusion:**

The identified risk factors are similar across the world; however, the suicide rates are not as high in other Western cultures as they are in South Korea. Future studies could compare Western countries to hierarchical countries to identify if there are any local risk factors that can help guide local prevention policies and educational programs with city officials’ engagement. Moreover, it would be essential to investigate the impact of stigma thoroughly since it is still hard to clarify whether it is a cultural issue or a worldwide issue preventing individuals from getting professional help.

